# Biochemical and Biophysical Characterization of Recombinant Yeast Proteasome Maturation Factor Ump1

**DOI:** 10.5936/csbj.201304006

**Published:** 2013-09-10

**Authors:** Bebiana Sá-Moura, Ana Marisa Simões, Joana Fraga, Humberto Fernandes, Isabel A. Abreu, Hugo M. Botelho, Cláudio M. Gomes, António J. Marques, R. Jürgen Dohmen, Paula C. Ramos, Sandra Macedo-Ribeiro

**Affiliations:** a IBMC - Instituto de Biologia Molecular e Celular, Universidade do Porto, Rua do Campo Alegre 823, 4150-180 Porto, Portugal; b Centre for Molecular and Structural Biomedicine, CBME/IBB, LA, Portugal; c Instituto de Tecnologia Química e Biológica, Universidade Nova de Lisboa, Portugal; d Institute for Genetics, University of Cologne, Zülpicher Str. 47, D-50674 Cologne, Germany; † Current address: Biophysics Section, Department of Life Sciences, Imperial College, London, UK; # Current address: BioFIG - Centre for Biodiversity, Functional and Integrative Genomics, Faculty of Sciences, University of Lisboa, Campo Grande 1749-016 Lisboa, Portugal; ‡ Current address: Immunodiagnostic Systems, Core Technology, 10 Didcot Way, Boldon, NE35 9PD, UK

**Keywords:** Circular dichroism, protein structure, dynamic light scattering, intrinsically disordered

## Abstract

Protein degradation is essential for maintaining cellular homeostasis. The proteasome is the central enzyme responsible for non-lysosomal protein degradation in eukaryotic cells. Although proteasome assembly is not yet completely understood, a number of cofactors required for proper assembly and maturation have been identified. Ump is a short-lived maturation factor required for the efficient biogenesis of the 20S proteasome. Upon the association of the two precursor complexes, Ump is encased and is rapidly degraded after the proteolytic sites in the interior of the nascent proteasome are activated. In order to further understand the mechanisms behind proteasomal maturation, we expressed and purified yeast Ump in E. coli for biophysical and structural analysis.

We show that recombinant Ump is purified as a mixture of different oligomeric species and that oligomerization is mediated by intermolecular disulfide bond formation involving the only cysteine residue present in the protein. Furthermore, a combination of bioinformatic, biochemical and structural analysis revealed that Ump shows characteristics of an intrinsically disordered protein, which might become structured only upon interaction with the proteasome subunits.

## Introduction

The 26S proteasome is the central player in the degradation of proteins by the ubiquitin-mediated proteolytic pathway in eukaryotic cells [1, 2]. This ATP-dependent multimeric protease complex is composed of the catalytic core particle (CP), named 20S proteasome that can be capped in one or both ends by the 19S regulatory particle. Recently, the topology of the 26S proteasome was revealed by electron microscopy studies [3, 4, 5]. Earlier crystallographic studies had provided a detailed knowledge of the subunit arrangement of the 20S CP [6, 7]. The eukaryotic CP is composed of 14 distinct subunits, seven α and seven β subunits, organized in four stacked heptameric rings with an α_1-7_β_1-7_β_1-7_α_1-7_ arrangement. The proteolytic sites are sequestered in the internal part of the chamber formed by the two β rings [6, 7, 8]. The active 20S CP is the result of a highly coordinated assembly process involving several conserved and specific chaperones. The dimeric chaperones Pba1-Pba2 and Pba3-Pba4 (‘Pba’, ‘proteasome biogenesis-associated’; also known as ‘Poc1-Poc4’ for ‘Proteasome chaperone’,) are thought to promote assembly of rings composed of seven α subunits, the platform for binding of the β subunits [9]. Ump1 (ubiquitin-mediated proteolysis), originally identified in *Saccharomyces cerevisiae*, was the first proteasome-specific assembly chaperone to be described [10]. Ump1, a ∼17 kDa short-lived protein, is found in precursor (or 15S) complexes called half-proteasomes that contain unprocessed β subunits [10, 11]. Besides Ump1, the 15S complexes contain all CP subunits, except for β7, and the chaperone Pba1-Pba2 [12, 13, –14]. Dimerization of two such complexes is triggered by incorporation of β7, whose C-terminal extension reaches out into the other half to stabilize the newly formed 20S particle [14, 15]. Ump1 is encased during precursor dimerization and is important for autocatalytic processing of β1, β2 and β5. Upon maturation of the active sites, Ump1 is rapidly degraded as the first substrate of the newly formed 20S CP [10, 16].

*S. cerevisiae* Ump1 is a conserved protein found in all eukaryotes [11]. The human ortholog, hUmp1, was shown to perform similar functions to its *S*. *cerevisiae* counterpart in proteasome biogenesis [17, 18, 19, 20, 21]. In contrast to yeast Ump1, hUmp1 appears to be essential for viability, as suggested by siRNA knockdown experiments [19, 20]. It was reported that hUmp1 binds to membranes leading to precursor proteasome assembly at the endoplasmic reticulum [22]. Studies in *S. cerevisiae* suggested that Ump1-containing precursor complexes are imported into the nucleus where the assembly of nuclear proteasomes is completed [23].

At present, no three-dimensional structure of Ump1 proteins is available, although there is some information on their functional domains. The C-terminal region, encompassing residues 51-147 of yeast Ump1 or 61-141 of human Ump1, is required and sufficient for interaction with proteasome precursor complexes. Residues 68 to 72 of hUmp1 are essential for this interaction [17]. By contrast, the region containing the first 50 amino acid residues of Ump1 is neither sufficient nor required for incorporation of Ump1 into precursor complexes [17]. These Ump1 regions are likely to operate by interacting with distinct substructures of different proteasome subunits. Indeed, hUmp1 binds directly to several α and β subunits and associates with α rings *in vitro* [19, 22, 24]. In line with this finding, hUmp1 appears to be essential for the binding of the β2 subunit to α ring precursor complexes, and therefore for the initiation and assembly of β rings [24]. *In vitro* experiments showed that hUmp1 binds directly to the β5 subunit [24]. Interestingly, the yeast β5 propeptide becomes dispensable in cells lacking Ump1, but is essential for viability in its presence [10, 25]. *In vivo* depletion of hUmp1 by siRNA experiments, however, prevented the incorporation of β5 into nascent proteasome precursor complexes [19].

Here we report the biochemical and biophysical characterization of recombinant yeast Ump1. Ump1 purified as a heterogeneous mixture of monomers and dimers. Dimer formation is mediated by Cys115. Mutation of this single cysteine to serine abolished dimer formation leading to preparations enriched in monomeric Ump1. Nevertheless, the purified mutated monomers were conformationally too heterogeneous to crystallize. A comparative biophysical analysis showed that Ump1 displays characteristics of a natively disordered protein. This biophysical property is independent of the oligomeric state of the protein and suggests that Ump1 structure might be stabilized upon interaction with proteasomal subunits and concomitant incorporation into proteasomal precursor complexes.

## Materials and methods

### Protein expression and purification

The plasmid pJD492-UMP1 was designed to express *UMP1-6xHis* yielding a non-cleavable C-terminally 6His-tagged version of *S. cerevisiae* Ump1. A PCR fragment, containing the nucleotide sequence of the complete UMP1 ORF, was cloned into pET11a using XbaI and BamHI restriction sites. The plasmid encodes the full-length Ump1 followed by the additional amino acid sequence GYHHHHHH. This plasmid was used as a template for construction of the mutant plasmid pJD492-UMP1-C115S by PCR. The following primers were synthesized to introduce the mutated sequence: 5′-CTA CTG AAC AAA GAG **TCC** AGC ATC GAT TGG GAG-3′ and 5′-CTC CCA ATC GAT GCT **GGA** CTC TTT GTT CAG TAG -3′ (bold represents mutation site). The mutation was confirmed by sequencing (Eurofins). Both plasmids encoding the 6His-tagged versions of yeast Ump1 under the control of a T7 promotor were used to transform *E. coli* BL21 CodonPlus (Stratagene) competent cells.

For expression of *S. cerevisiae* Ump1 and Ump1-C115S, *E. coli* BL21 CodonPlus (Stratagene) transformed with the expression plasmids were grown in lysogeny broth (LB) medium containing ampicillin and chloramphenicol (final concentrations of 100 µg/mL and 34 µg/mL, respectively), and incubated at 37°C until OD_600_ reached approximately 0.3. The incubation temperature was reduced to 24 °C, before induction of protein expression by addition of IPTG (Biosynth) to a final concentration of 2 mM. Cells were harvested by centrifugation 4 h after induction and the cell pellet from each liter of culture was resuspended in 20 mL of lysis buffer (0.1% (v/v) Tween 20, 300 mM NaCl, 10 mM imidazole in PBS (phosphate buffered saline – 137 mM NaCl, 2.7 mM KCl, 10 mM Na_2_HPO_4_, 4 mM KH_2_PO_4_, pH 8.0) supplemented with 50 µg/mL of lysozyme and stored at -20 °C. Upon thawing, complete EDTA-free protease inhibitor cocktail (Roche), 5 µg/mL DNAse I and 10 mM MgCl_2_ (final concentration) were added to the cell lysate, which was centrifuged and the supernatant loaded onto a 5 mL HisTrap column (GE Healthcare) previously equilibrated with buffer A (20 mM sodium phosphate pH 8.0, 500 mM NaCl and 10 mM imidazole). The column was washed with 10 column volumes of buffer A and bound proteins were eluted with 100 mM imidazole in buffer A.

Fractions containing recombinant Ump1 were pooled, desalted on a HiPrep 26/10 column (GE Healthcare), previously equilibrated in 50 mM Tris-HCl pH 7.5. The desalted Ump1 fraction was further purified on an anion-exchange column (MonoQ; GE Healthcare), using a linear 0 to 1 M NaCl gradient in 50 mM Tris-HCl pH 7.5. The oligomeric state of the protein was verified by size-exclusion chromatography on a Superdex 75 column (GE Healthcare) equilibrated with 50 mM Tris pH 7.5, 100 mM NaCl. The column was calibrated using aprotinin (6.5 kDa), ribonuclease A (13.7 kDa), chymotrypsinogen (25.0 kDa), and ovalbumin (43.0 kDa) as standards. The void volume (Vo) was calculated by determining the elution volume of dextran blue. The partition coefficient (Kav) for each protein was obtained with the following equation: Kav=(Ve-Vo)/(Vt-Vo), where Ve is the elution volume and Vt is the total bed volume. A standard calibration curve of Kav versus log(MW) was used to calculate the apparent molecular mass of the distinct recombinant Ump1 molecular species. The Stokes radius (Rs) for the globular protein standards was calculated with the equation Log(Rs) = -(0.204±0.023)+(0.357±0.005)•log(MW) [26]. These values were used to create a calibration curve (1000/Ve vs. Rs), which allowed the determination of the Rs for the distinct Ump1 molecular species. For plotting the theoretical relationship between Rs and MW for proteins in native (Native), natively unfolded pre-molten globule (nu-PMG) and urea-unfolded (un) conformations, the following equations were used: log(Rs^Native^) =-(0.204 ±0.023)+(0.357 ± 0.005)•log(MW); log(Rs^nu-PMG^) =-(0.239 ±0.055)+(0.403 ± 0.012)•log(MW) and log(Rs^un^) =-(0.723 ±0.033)+(0.543 ± 0.007)•log(MW) [26].

### Dynamic Light-Scattering (DLS) measurements

Hydrodynamic radius (RH) measurements were made at 25°C with a Zetasizer Nano ZS DLS apparatus (Malvern Instruments). A sample (50 µl) containing 0.5 mg/ml protein in 50 mM Tris-HCl pH 7.5, 100 mM NaCl was centrifuged and filtered through a 0.2 µm filter to remove suspended particles, and placed in a quartz cuvette. Particle diffusion coefficients were calculated from auto-correlated light intensity data, and converted to RH with the Stokes-Einstein equation (*D*_*t*_ = *k*_*B*_ *T*/6πη*R*_*H*_, where *k*_*B*_ is the Boltzmann constant; *T* is temperature in Kelvin; η is solvent viscosity; and *R*_*H*_ is the hydrodynamic radius of the protein). A histogram of the percentage of the scattering mass versus RH was calculated using DTS (nano) 6.01 software (Malvern Instruments). Data represent an average of 3 measurements for each sample.

### Limited proteolysis

Limited proteolysis assays were performed by incubating the purified recombinant protein with trypsin at a ratio of 1000:1 (w/w) in 50 mM Tris-HCl pH 8.0 and 100 mM NaCl, at 37°C. Aliquots were collected at specific time points (0 and 30 min) and reactions were stopped by incubation at 95°C for 5 minutes in standard sample buffer without or with 10 mM DTT. The cleavage products were separated by SDS-PAGE (17.5% acrylamide gel), transferred onto PVDF membrane, and analysed by Edman degradation.

For analysis of the secondary structure content of the N- and C- terminal peptides, recombinant Ump1-C115S was treated with trypsin for 30 min and the solution obtained after limited proteolysis was applied to a 1 mL HisTrap column (GE Healthcare) previously equilibrated with buffer A, and the unbound N-terminal fragment collected by washing with 2 column volumes of buffer A. The bound proteins were eluted with 100 mM imidazole in buffer A and contained a mixture of full-length Ump1 and the His-tagged C-terminal peptide. The purified N-terminal fragment was dialysed against 50 mM Tris-HCl pH 7.5, 100 mM NaCl, concentrated to 5 mg/mL and used for CD analysis.

### Circular dicroism

The secondary structure content of full-length Ump1 was assessed by far-UV circular dichroism (CD) spectroscopy. Measurements were performed on a Jasco J-815 spectrometer equipped with a Peltier-controlled thermostated cell support. Ump1 solutions were 0.1 mg/ml in 50 mM Tris-HCl pH 7.5, 100 mM NaCl with or without 1 mM DTT (freshly prepared and incubated for 1 h at 4°C). CD spectra were acquired at 25°C, with the instrument set up to 2 nm bandwidth, 1 s response, 200 nm/min scanning speed and 10 accumulations. Spectra were deconvoluted with CDNN 2.1 [27]. Thermal unfolding was performed by raising the temperature at a rate of 1 °C/min, between 25 and 90°C, while monitoring the CD signal at 205 nm. The unfolded protein fraction was calculated by normalizing the CD signal variation.

For analysis of the secondary structure content of Ump1-C115S and N-terminal fragment by far-UV CD in buffer without DTT and low NaCl concentration, the proteins were diluted to a final concentration of 0.1 mg/mL in 1 mM Tris-HCl pH 7.5, 2 mM NaCl, and measurements were performed at 20°C on a Jasco J-815 spectrometer fitted with a Peltier temperature controller. Spectra were acquired between 190 and 260 nm, set up to 1 nm bandwidth, 1 s response, 500 nm/min scanning speed and 3 accumulations. Each spectrum was the average of two scans corrected for buffer background. The spectra were deconvoluted with the CONTIN program using the online software Dichroweb [28, 29].

### Analysis of primary sequence and prediction of disorder

Prediction of disorder for Ump1 was performed on multiple sequence alignments with RONN (http://www.bioinformatics.nl/∼berndb/ronn.html) that uses a modification of the Bio-Basis Function Neural Network (BBFNN) [30] and Fold Index [31], based on the algorithm of Uversky and coworkers [32]. For comparison with other available disorder prediction servers yeast Ump1 sequence was also analysed with the Meta Protein DisOrder prediction System (http://prdos.hgc.jp/cgi-bin/meta/top.cgi), an online webserver that predicts the disorder tendency of each residue resorting to the prediction results of the seven independent disorder predictors [33] (Figure S2).

## Results

### Ump1 purified under non-reducing conditions self-assembles into oligomeric species

**Figure 1:**
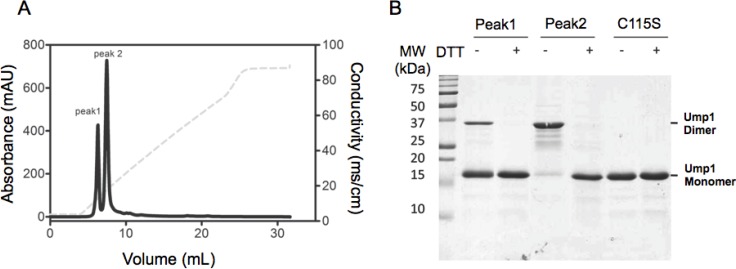
**Recombinant Ump1 is purified as a mixture of molecular species with different charges and oligomeric states**. A) Ion-exchange chromatographic profile of the metal-affinity purified Ump1 fraction shows that this protein is further separated into two peaks corresponding to species with different isoelectric points (peak 1 and peak 2). Conductivity is represented by a dotted line. B) Electrophoretic analysis of Ump1 fractions corresponding to peak 1 (monomer) and peak 2 (dimer) of the ion-exchange chromatography. The wild-type Ump1 monomer is frequently contaminated with dimers under non-reducing conditions (first lane). The Ump1-C115S mutant elutes from the ion-exchange column as a single peak (data not shown) and migrates as the wild-type Ump1 monomer. Proteins were loaded in sample buffer without (-) or with (+) 10 mM DTT prior to electrophoresis in a 15% SDS-PAGE (here stained with Coomassie Blue). MW, Molecular weight marker; values in kDa.

Yeast Ump1, expressed in *E. coli* and containing a C-terminal 6His tag, was efficiently purified by metal affinity chromatography. In a subsequent ion-exchange chromatography, two Ump1-containing peaks were eluted with different NaCl concentrations (Figure 1A). This elution profile and isoelectric focusing (data not shown), indicated that recombinant Ump1 purified by metal affinity chromatography was heterogeneous and contained at least two differently charged species. Analysis by SDS-PAGE showed that, under reducing conditions, the proteins eluting in the different peaks after ion exchange chromatography were indistinguishable (Figure 1B). However, when no reducing agent was added, the protein eluting with lower NaCl concentration migrated faster (apparent MW 18 kDa corresponding to the predicted value for the tagged protein, and from here on referred to as monomer, Figure 1) than the protein eluted with higher NaCl concentrations (apparent MW 36 kDa and from here on referred to as dimer, Figure 1). Taken together these data indicated that Ump1 was purified as a mixture of presumably monomers and dimers (under non-reducing conditions), and that self-association was mediated by formation of an intermolecular disulfide bond.

### A cysteine residue at position 115 is involved in Ump1 oligomerization

**Figure 2:**
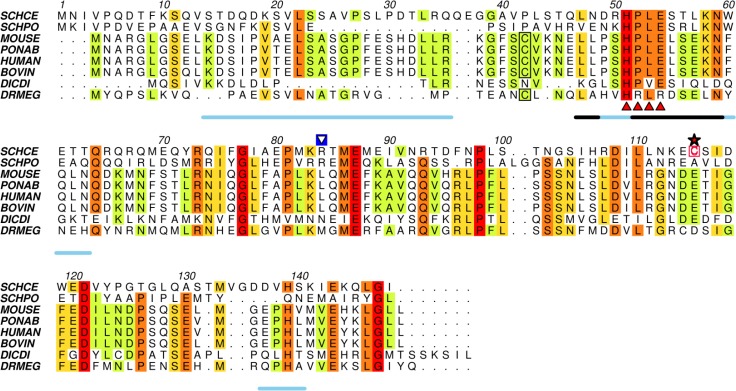
**The N-terminal half of Ump1 is predicted to be highly disordered**. Amino acid sequence alignment of selected Ump1 proteins (see Table S1 for protein% similarities) was performed with ClustalW2, and rendered with Aline [35]. Disorder was predicted with RONN [30] for the selected amino acid sequences and a consensus line for disorder prediction (http://www.bioinformatics.nl/∼berndb/ronn.html) is printed below the alignment: the black line highlights residues where disorder is predicted for all the displayed sequences and the blue line represents regions where disorder is predicated for least 80% of the represented Ump1 orthologs. The position of the non-conserved Cys115 is indicated by a red star, the conserved motif HPLE is indicated by red triangles, and the Cys37 residue conserved in mammalian orthologs is boxed. The blue-boxed arrow above Arg84 points to one of the trypsin-cleavage sites identified by N-terminal sequencing after limited proteolysis experiments (Figure S1). SCHCE, Ump1 from *Saccharomyces cerevisiae* (UniProt accession code P38293); SCHPO, Ump1 from *Schizosaccharomyces pombe* (O74416); MOUSE, Ump1 from *Mus musculus* (Q9CQT5); PONAB, Ump1 from *Pongo abelii* (Q5R9L9); HUMAN, Ump1 from *Homo sapiens* (Q9Y244); BOVIN, Ump1 from *Bos taurus* (Q3SZV5); DICDI, Ump1 ortholog from *Dictyostelium discoideum* (Q55G18) and DRMEG, Ump1 from *Drosophila melanogaster* (Q9VIJ5).

Analysis of the Ump1 amino acid sequence (Figure 2) shows that disulfide bond formation likely involves the single non-conserved cysteine residue at position 115. Interestingly, previous work with the recombinant human Ump1 ortholog revealed that it also self-assembles and that oligomerization is likely to be mediated by a cysteine residue (Cys37) located in the N-terminal region of the protein [34].

**Figure 3:**
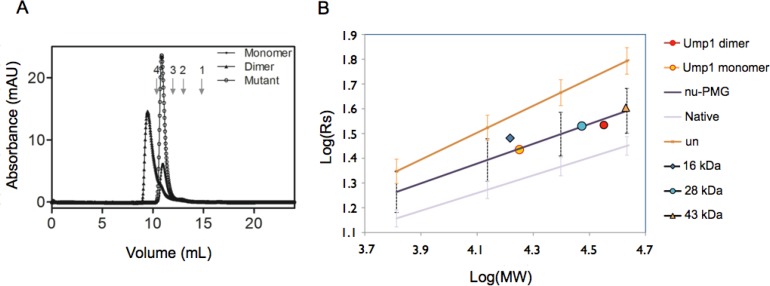
**Determination of Ump1 apparent molecular mass and Stokes radii (Rs)**. A) Size-exclusion chromatography of wild-type Ump1 dimer and Ump1-C115S monomer. Superdex 75 calibration was performed with the following molecular weight protein standards: 1 - aprotinin (6.5 kDa), 2 – ribonuclease A (13.7 kDa), 3 - chymotrypsinogen (25.0 kDa), and 4 - ovalbumin (43.0 kDa). Ump1 wild-type dimer and C115S monomer display atypical mobility, eluting with apparently higher molecular masses of 65 and 40 kDa, respectively (calculated with the equation Kav = -2.0693•log(MW) + 4.9698, R2 = 0.99607, obtained after column calibration). Using these data, the apparent Rs calculated for wild-type Ump1 dimer and C115S monomer are 34 and 27 Å, respectively (as calculated from the equation Rs = 0.3467(1000/Ve)-5.7834, R2 = 0.99061; Ve = elution volume). B) Logarithmic plot of Rs versus molecular mass (MW) of the corresponding proteins. The straight lines represent the average theoretical Rs for the proteins used as standards, assuming different conformational states (native), a natively unfolded pre-molten globule-like conformation (nu-PMG) or a non-native urea-denatured conformation (un) according to the equations given in ref [26]. The error bars represent the standard deviation for each plot as calculated from ref. [26]. Ump1 monomer (C115S) and Ump1 dimer correspond to the orange and red circles, respectively and fall within the range of values expected for natively unfolded molten globule conformation. For comparison purposes, experimentally determined values for Rs [36] are shown for pre-molten globule conformations of proteins with molecular masses of 43 kDa (MMP-1 Interstitial collagenase, orange triangle), 28 kDa (Tryptophan synthase, blue circle) and 15 kDa (Tumor suppressor p16, blue rhombus).

Analysis of the two peaks obtained by size exclusion chromatography (Figure 3) confirmed that the two Ump1 fractions correspond to different oligomeric states of the recombinant protein. Purification under reducing conditions (addition of 1-5 mM DTT in all chromatography and protein storage buffers) increased the yield of the Ump1 species with lower molecular weight (monomer, data not shown), but this protein slowly converted to a mixture of the two forms, rendering this sample too heterogeneous for further biophysical and structural studies.

In an attempt to obtain homogeneous protein, and to confirm the implication of cysteine 115 in Ump1 dimerization, we mutated this residue to a serine. The purified Ump1-C115S mutant was analyzed by SDS-PAGE (Figure 1B), size-exclusion chromatography (Figure 3), and DLS (Table 1) and compared to wild-type Ump1 purified under non-reducing conditions. The mutant protein purified as a single peak in the ion-exchange column (data not shown) and in the analytical size exclusion chromatography (Figure 3A). The C115S mutant eluted with a lower apparent molecular weight than that of the wild-type disulfide-bonded Ump1 dimer, supporting the hypothesis that Cys115 is responsible for the oligomerization of wild-type Ump1. However, both Ump1 species eluted with apparent molecular masses (40 and 65 kDa for the lower and higher molecular mass Ump1 species, respectively) that are larger than the theoretical values for monomeric (18 kDa) or dimeric (36 kDa) tagged Ump1. The apparent molecular mass determined for the lower molecular weight species, is larger than a monomer and approaches the value expected for a non-covalently associated dimer. Similarly, the higher molecular mass species displays an intermediate size, closer to a tetramer. Since this atypical mobility is a characteristic of intrinsically disordered proteins [37], one hypothesis to support these results is that the purified Ump1 species could represent a mixture of monomers (with identical elution profiles to Ump1-C115S) and covalently associated dimers (wild-type Ump1 higher molecular mass species) with non-compact elongated shapes, resulting in anomalous migration in size-exclusion chromatography.

**Table 1:** Ump1 hydrodynamic radius as determined by dynamic light scattering.

Ump1 species	Polydispersity index	Stokes radius (Å)	Calculated molecular mass (kDa)	Percentage of scattering volume in solution
**Ump1 wild-type monomer**	0.3	18.1	13.5	15.1%
		20.9	18.9	17.4%
		24.2	26.6	16.4%
**Ump1 wild-type dimer**	0.3	24.2	26.6	15.5%
		28	37.4	17.4%
		32.5	53.1	16.1%
**Ump1-C115S monomer**	0.2	20.9	18.9	15.7%
		24.2	26.6	16.9%
		28.1	39	15.4%

The calculation of the Stokes radii, which was based upon the values of a standard calibration curve (Figure 3) revealed values of 27 Å and 34 Å for the lower and higher molecular mass species, respectively. To obtain another estimate of the hydrodynamic dimensions of the protein in solution, the diffusion coefficient was measured by dynamic light scattering (DLS). All samples have high polydispersity indices, and show a heterogeneous distribution of particles with different molecular sizes in solution (Table 1), with ∼50% of the scattering volume attributed to particles ranging between 18 and 24 Å for the monomeric wild-type Ump1, and between 24 and 32 Å for dimeric Ump1. These data reinforce the view that both recombinant wild-type Ump1 and the C115S mutant are highly heterogeneous in solution.

The logarithmic plot of these calculated Rs values versus the molecular masses of the corresponding monomeric and dimeric Ump1 variants indicates that these proteins do not behave as natively folded globular proteins in solution, and fall very close to the plot representing the behaviour of molecules with a natively unfolded molten globule conformation (Figure 3B). All results indicate that the Rs for the recombinantly expressed Ump1 molecular species are significantly larger than expected for a globular protein of similar molecular mass. Despite the current experimental evidence, however, it cannot be excluded that non-covalent oligomerization is a reason for the higher-than-predicted apparent molecular masses of the monomeric and dimeric Ump1 species in solution. The data suggest that this protein is at least partially unfolded and alternates between multiple extended conformations with variable hydrodynamic radius. In addition, the C115S mutation, although eliminating the heterogeneity attributed to the formation of covalently associated wild-type Ump1 oligomers, did not prevent the appearance of molecules with variable sizes as clearly seen in the DLS data (Table 1), and likely attributable to conformational variation between slightly more compact and extended conformations.

### Recombinant Ump1 is partially disordered in solution

In agreement with the hypothesis that Ump1 is at least partially unfolded, leading to the apparently higher hydrodynamic radius of the different molecular species of recombinant Ump1, analysis of its primary sequence shows that 33% of its amino acid residues are predicted to be disordered (Figure 2). These residues are mainly distributed in the N-terminal half of the protein, comprising amino acids 12-38 and 47-63 (Figure 2 and S2). The prediction of disorder extends to the sequences of Ump1 orthologs, indicating that the regions predicted to be partially unfolded might have a functional significance.

**Figure 4:**
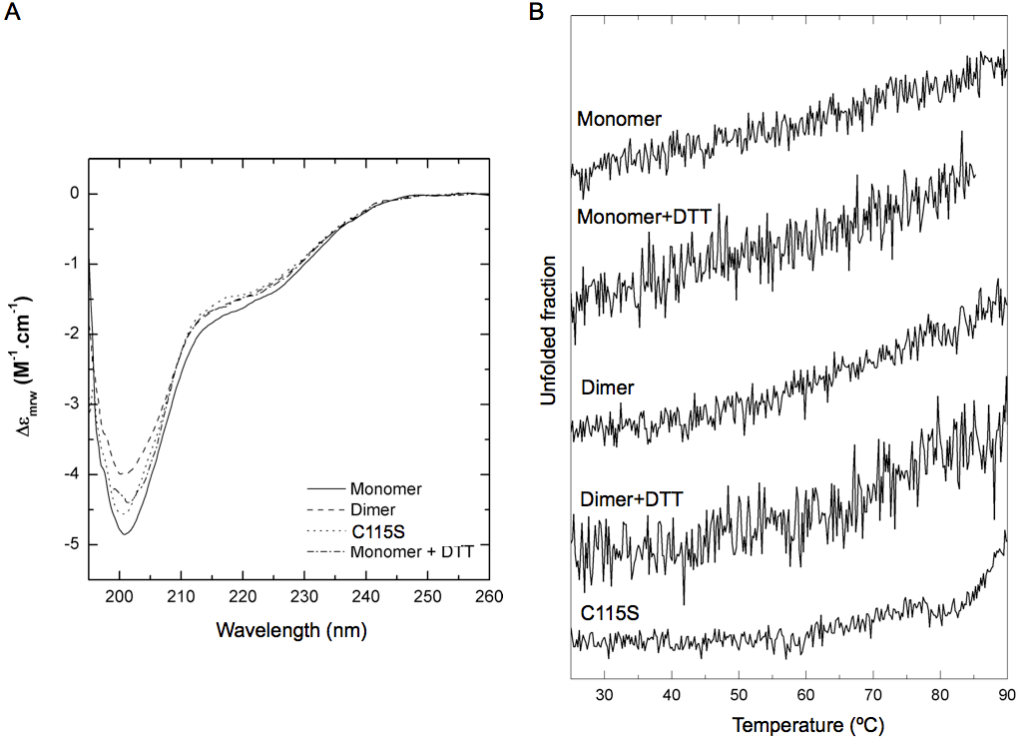
**Analysis of Ump1 secondary structure and conformational stability by circular dichroism**. A) Far-UV CD spectra of wild-type (monomer and dimer) and mutant Ump1. Monomeric wild-type Ump1 was prepared in the presence or absence of freshly prepared 1mM DTT. The strong negative peak at 201 nm is characteristic of abundant random coil structures. B) Denaturation curves were computed for wild-type (monomer and dimer, both in the presence and absence of DTT) and Ump1-C115S from the CD variation at 205 nm ellipticity (mdeg). Higher (i.e. less negative) signals indicate lower structural content and unfolding. Non-cooperative unfolding and resistance to full denaturation are characteristic of proteins harbouring unstructured segments [38].

Circular dichroism (CD) spectra were recorded to compare the secondary structure content of the wild-type and mutant Ump1 oligomeric species, and thus confirm its folding state. The CD spectra for all proteins (monomeric and dimeric wild-type Ump1, as well as Ump1-C115S mutant) exhibit isodichroic curves, with a minimum at 201 nm and a shoulder around 222 nm (Figure 4A). The negative peak is characteristic of random coil structures. Spectral similarity indicates similar secondary structure content in all Ump1 preparations. These results provide evidence for the presence of structured and unstructured regions in Ump1, in agreement with the disorder predictions. The secondary structure content, however, is not significantly affected by the oligomeric state of the protein or by the C115S mutation.

To gain complementary insight into the folding properties of Ump1, we performed thermal denaturation assays of monomeric and dimeric wild-type versions (in the presence or absence of DTT) as well as of the C115S mutant, while simultaneously monitoring the CD signal at 205 nm (Figure 4B). Interestingly, all preparations of wild-type Ump1 exhibit a very gradual – and almost constant – CD signal variation with temperature, from 25 to 90°C. This is unlike the typical behaviour of small, single domain folded globular proteins, where the unfolding is highly cooperative and occurs in a very narrow temperature range. Also, even at 90°C, Ump1 does not seem to be fully denatured, as seen by the fact that the CD signal does not plateau at high temperature. This behaviour is what one would expect from a protein harbouring unstructured regions, since the inability to maintain a compact hydrophobic core would, (i) hinder the establishment of the interaction network responsible for folding cooperativity, and (ii) substantially increase the conformational entropy and therefore increase the resistance to full unfolding. The C115S mutant exhibits higher unfolding cooperativity, but the overall considerations made for the wild-type still apply.

**Figure 5:**
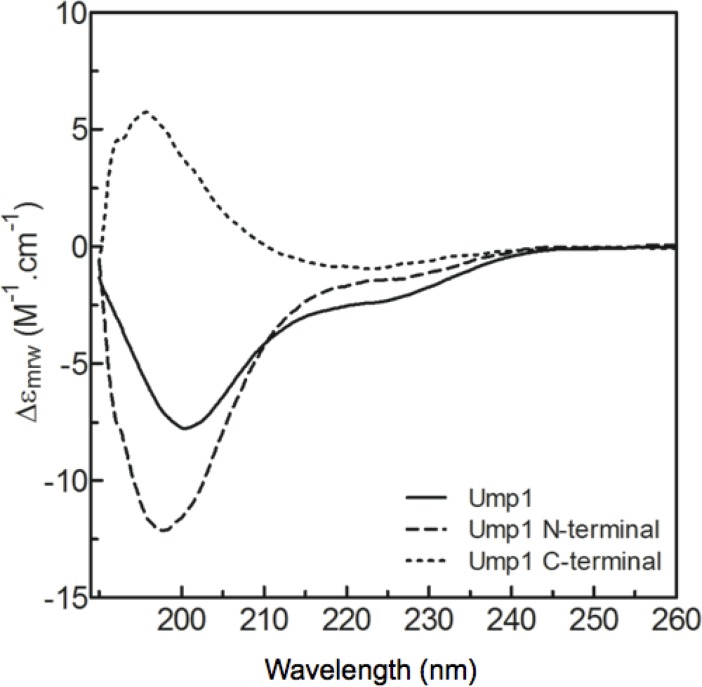
**The N-terminal region of Ump1 is highly disordered**. Far-UV CD spectra of Ump1-C115S and its isolated N-terminal fragment. The difference spectrum for the C-terminal peptide was obtained by subtracting the N-terminal Ump1 spectrum from that of full-length Ump1-C115S. Upon deconvolution the secondary structure of Ump1 N-terminal is 1% α-helix, 25% β-strand, 22% turns and 50% coil. The C-terminal peptide secondary structure corresponds to 18% α-helix, 37% β-strand, 19% turns and 27% coil.

Limited proteolysis experiments shows that Ump1 is cleaved by trypsin at Arg84 (Figure S1), leaving an N-terminal fragment that includes most of the region predicted to be unstructured as well as the conserved HPLE sequence (Figure 2) required for proteasome interaction [17]. The CD spectra from this Ump1 N-terminal proteolytic fragment confirm that, in accordance with the theoretical disorder predictions (Figure 2 and S2), the N-terminal region is largely unstructured (Figure 5). Spectral deconvolution of the full-length Ump1-C115S reveals that it contains 19% α-helices, 20% β-strands, 19% turns and around 42% random coil. The N-terminal segment is predominantly composed of random coil (∼50%), with 24% of β-strands, 22% turns and a negligible amount of α-helices (∼1%). The C-terminal spectrum, obtained by subtracting the N-terminal Ump1 spectrum from that of Ump1-C115S, provides an estimate of the secondary structure content of the C-terminal region and suggests that this region has a significant secondary structure content with a relatively lower percentage of coil regions (27% coil, 17% α-helices, 37% β-strands and 19% turns).

## Discussion

Biochemical characterization of *S. cerevisiae* Ump1 was performed using a variety of techniques. The results obtained by size exclusion chromatography and CD, together with amino acid sequence analysis, show that recombinant Ump1 is a natively unfolded protein. Accurate identification of these disordered regions in proteins, which confer conformational heterogeneity to the samples but are often mediators of protein-protein interactions, is crucial for structural and functional studies.

The recombinantly expressed and purified Ump1 consists of a heterogeneous mixture of molecules with variable isoelectric points and hydrodynamic radii. In particular, the non-conserved single cysteine at position 115 is partly responsible for this heterogeneity leading to Ump1 self-assembly by disulfide-bond formation. Conceivably, dimerization may play a role in proteasome biogenesis, a process that could be modulated by the local redox state of the cell. Indeed, disulfide-mediated virion assembly in the cytosol catalyzed by virus-encoded redox-regulated proteins has been previously demonstrated [39]. However, the lack of evolutionary conservation of this cysteine residue (Figure 2) may indicate that cysteine-mediated dimerization might not have a key role in Ump1 function *in vivo*. Mutation of Cys115 to serine eliminates the formation of covalently associated Ump1 oligomers, but the anomalously large Stokes radius of this monomeric form suggests that the protein is not globular and its conformation is predicted to be a natively unfolded molten globule.

The intrinsic disorder of Ump1 is supported by CD analysis of the secondary structure content, which indicated that ∼42% of its structure is dominated by a random coil conformation (Figure 4A, Figure 5). These data are in agreement with a theoretical prediction of disorder, particularly relevant in the N-terminal half of the protein (Figure 2, Figure S2), which was shown to be ∼50% random coil (Figure 5). Moreover, the low unfolding cooperativity and high stability of Ump1 towards unfolding by temperature (Figure 4B) constitute additional fingerprints for structurally disordered proteins. In this context, it is worth noting that the Ump1 region 51-147 starting at the conserved HPLE motif, which is predicted to be unstructured (Figure 2), is sufficient for interaction with proteasome precursor complexes [17]. The flexibility of its N-terminal domain may give the protein the ability to bind multiple targets during proteasome assembly. One possibility is that the N-terminal region of Ump1 engages in interactions with components of a second 15S complex during their dimerization [16]. Another important aspect of a lack of regular secondary structure is that it might provide Ump1 with the capability to adjust to steric restrictions upon enclosure in the newly formed proteasome following dimerization of 15S precursor complexes [13].

There is a currently growing awareness of the fundamental importance of disordered regions of proteins in many biological and pathological processes [38, 40]. These regions, characterized by the absence of a well-defined three-dimensional structure and displaying structural flexibility, are highly abundant in eukaryotic proteomes. These features are proposed to provide a functional advantage to proteins by enabling them to interact with multiple binding partners and to behave as intracellular hubs [41]. The inherent plasticity of these intrinsically disordered regions allows them to play fundamental roles in macromolecular recognition and assembly, and to be active players in molecular events such as intracellular signalling, which require transient interactions and shuttling between different macromolecular assemblies.

Ump1 mechanism of action is not yet completely understood and its known interaction partners are limited to some proteasome subunits. Ump1 was proposed to provide a checkpoint that prevents early dimerization of precursor complexes until their assembly is completed [13]. The propeptides of proteasome subunits β5 and β6, as well as the β7 C-terminal extension might contribute to overcome this checkpoint after incorporation of β7 by displacing Ump1 or changing its conformation [10, 13, 16]. Structural flexibility of Ump1 might be a key characteristic enabling these adjustments.

Characterization of Ump1, a key factor in proteasome biogenesis, may open a window of opportunity for the development of new proteasome inhibitors. Since the proteasome has been shown to be a suitable target in cancer therapy [42], development of alternative or additional proteasome inhibitors that interfere with proteasome assembly might contribute enormously to cancer treatment.

## Supplementary Material

Biochemical and Biophysical Characterization of Recombinant Yeast Proteasome Maturation Factor Ump1
