# Supplementary material for: Biochemical and Biophysical Characterization of Recombinant Yeast Proteasome Maturation Factor Ump1
Source: Comput Struct Biotechnol J. 2013 Sep 10;7:e201304006. doi: 10.5936/csbj.201304006 (PMC3962104; doi:10.5936/csbj.201304006)
Supplement: Biochemical and Biophysical Characterization of Recombinant Yeast Proteasome Maturation Factor Ump1 [file CSBJ-7-e201304006_SM0001.pdf]

Supplementary Figures and Tables

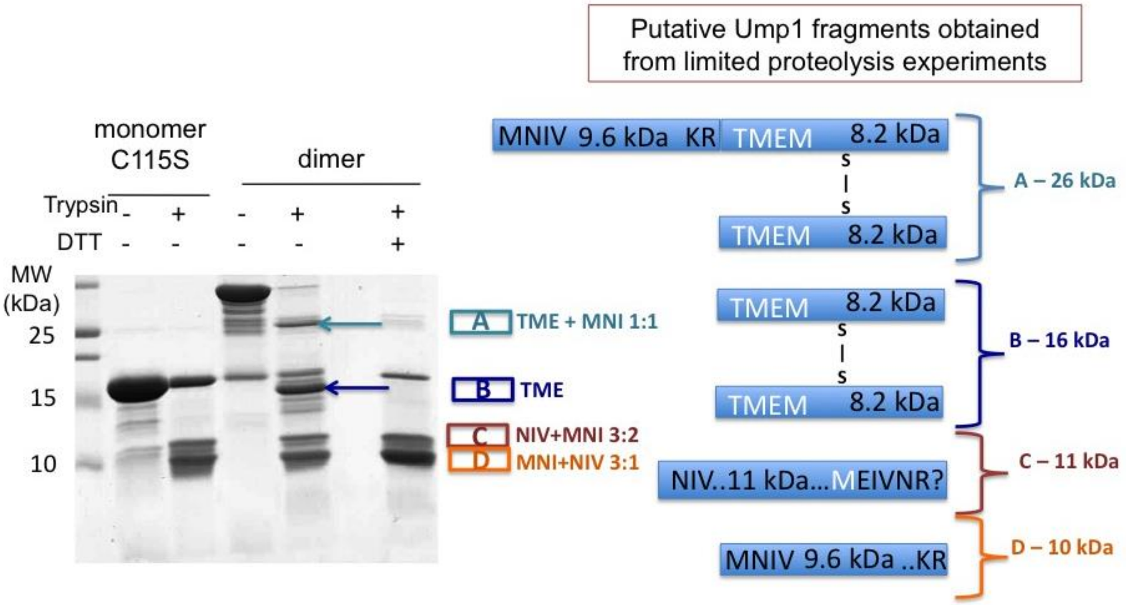

Figure S1 – Limited proteolysis of recombinant yeast Ump1. Ump1-C115S monomer and wild-type dimer were incubated with trypsin (w/w 1000:1) at 37 °C for 30 minutes and the proteolysis fragments were separated by SDS-PAGE. The selected fragments (A, B, C and D) were analyzed by N-terminal sequencing and the results are displayed on the right side of the SDS-PAGE image. The main cleavage site identified is Arg84, which originates a 9.6 kDa N-terminal fragment and a 8.2 kDa C-terminal peptide. The C-terminal fragment, starting with the TMEM sequence can only be identified in dimeric Ump1 before disruption of the disulfide bridge with DTT. This might indicate that this peptide alone displays anomalous migration or it could not be efficiently transferred onto the PVDF membrane before N-terminal sequencing. Based on the analysis of the protein sequence and on the relative MW of the proteolysis fragments a possible interpretation of the results is presented on the right side of the image.

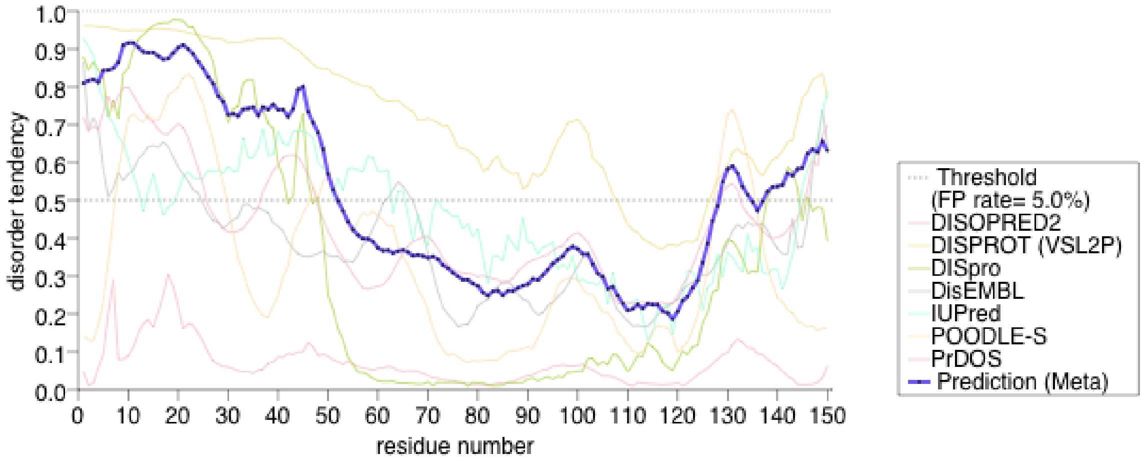

Figure S2 - Disorder profile plot showing the combined disorder tendency for each residue in the yeast Ump1 amino acid sequence, obtained with the Meta Protein DisOrder prediction System [33].

Table S1 – Similarity between the amino acid sequences of Ump1 orthologs selected for the alignment shown in Figure 2. The indicated values correspond to pairwise % of identity for the selected protein pairs.

PONAB	HUMAN	BOVIN	MOUSE	DRMEG	SCHCE	SCHPO	DICDI	
100	100	97	95	43	23	25	30	PONAB
	100	97	95	43	23	25	30	HUMAN
		100	96	43	25	25	30	BOVIN
			100	42	24	26	31	MOUSE
				100	27	23	20	DRMEG
					100	34	18	SCHCE
						100	20	SCHPO
							100	DICDI
